# Effects of pH on High-Performance ZnO Resistive Humidity Sensors Using One-Step Synthesis

**DOI:** 10.3390/s19235267

**Published:** 2019-11-29

**Authors:** Shuguo Yu, Hongyan Zhang, Jun Zhang, Zhijun Li

**Affiliations:** School of Physical Science and Technology, Xinjiang University, Urumqi 830046, China; yushuguo0818@163.com (S.Y.); lizhjun@xju.edu.cn (Z.L.)

**Keywords:** humidity sensor, zinc oxide, pH value, hydroxyl group, oxygen vacancies

## Abstract

In this paper, we prepared a high-performance zinc oxide (ZnO) humidity sensor in an alkaline environment using one-step hydrothermal method. Experiments showed that the pH value of the precursor solution affects the performance of ZnO humidity sensors. There are abundant hydroxyl group and oxygen vacancies on the surface of ZnO with a precursor pH value of 10. Abundant hydroxyl groups on the surface of ZnO can adsorb a large number of water molecules and rich oxygen vacancies can accelerate the decomposition of water molecules, thus increasing the number of conductive ions (H_3_O^+^) and further improving the performance of the sensor. So, such a ZnO humidity sensor exhibited high sensitivity (14,415), good linearity, small hysteresis (0.9%), fast response/recovery time (31/15 s) in the range from 11% to 95% relative humidity (RH). Moreover, the ZnO-2 humidity sensor has good repeatability and can be effectively used for a long time. This study provides a new idea for the development of low-cost, high-performance and reusable ZnO resistive humidity sensors.

## 1. Introduction

In recent years, humidity sensors have played an important role in the development of medical, food preservation, environmental monitoring, semiconductor, and other industries [1,2,3,4]. With the deepening of research, humidity sensors are required to meet special characteristics, such as high sensitivity, fast response/recovery speed, low hysteresis, and long-term stability [5,6,7]. Sensitive materials are a major factor to improve the performance of humidity sensors due to their different chemical structures and special functions. So, the development of sensitive materials has become a hot-spot to improve humidity sensitivity. In general, ceramics, semi-conductive metal oxides, polymers, and carbon-based nanomaterials are the most common sensitive materials [8,9,10,11]. Among them, metal oxide semiconductor materials such as ZnO, TiO_2_, CuO, and Al_2_O_3_ are widely used for sensor development due to their simple fabrication, high sensing response, good operational stability, low cost, and good portability [12,13,14,15]. Among them, ZnO has become widely studied as a nano-sensing material because it is plentiful and has controllable surface morphology, wide resistivity range, good chemical stability, and thermal stability [15,16].

Recently, some studies have found that specific surface area, electrical conductivity, and surface oxygen vacancies have become important factors to determine the performance of ZnO humidity sensors [17]. However, the existing ZnO has a low specific surface area and electron mobility and lack of hydrophilic functional groups on its surface, which severely limits its development in the field of humidity sensors [18,19]. Thus, the synthesis of ZnO with rich oxygen vacancies, hydrophilic functional groups, high specific surface area, and high electron mobility has become one of the key problems in humidity sensors [20]. There are many methods to solve the above problems and further improve the performance of ZnO humidity sensors, including ion doping, modification, and recombination [21,22]. However, these methods have complicated synthesis processes, are difficult to control, and have high raw material prices, which greatly increases the production costs. Furthermore, there are few studies on improving the humidity sensing performance by controlling the synthesis conditions of ZnO. It is well known that synthesis in an alkaline environment increases the surface oxygen vacancies and the concentration of hydroxyl groups (–OH) in ZnO. The more oxygen vacancy defects there are on the surface, the more active the adsorption of water molecules. Hence, water molecules on the surface of ZnO can produce a large amount of conductive H_3_O^+^ [23]. The hydroxyl group (–OH) is a hydrophilic functional group, which can adsorb more water molecules, thereby improving the performance of the sensor [24]. The synthesis of ZnO in an alkaline environment provides a new idea to improve the performance of ZnO humidity sensors.

In the present study, a ZnO resistive humidity sensor is synthesized by the one-step hydrothermal method. The experimental results show that the alkaline environment is more suitable to increase the concentration of oxygen vacancies on the surface of ZnO, which can improve the performance of the sensor by accelerating the decomposition of water molecules. In addition, the hydrophilic functional group (OH^−^) on the surface of ZnO is one of the main reasons to enhance the performance of the sensor, and it can adsorb a large amount of water molecules. Such a ZnO humidity sensor exhibits high sensitivity, good linearity, small hysteresis, and fast response/recovery time in the range of 11% to 95% RH. Therefore, the ZnO sensitive material is expected to develop a low-cost, high-performance, and reusable humidity sensor.

## 2. Materials and Methods

### 2.1. Materials and Synthesis

Zinc acetate (Zn(CH_3_COO)_2_·2H_2_O), sodium citrate (Na_3_C_6_H_5_O_7_·2H_2_O), sodium hydroxide (NaOH), and ethanol (AR) were purchased from Sangon Biotech (China, www.sangon.com). ZnO was synthesized by a simple one-step method. A total of 0.2195 g of zinc acetate and 0.2941 g of sodium citrate were dissolved in 40 mL of deionized water and magnetically stirred for 10 min to form a clear solution. The above mixed solution was used as a precursor solution. Then, a certain amount of 3 mol/L NaOH solution was added dropwise to the precursor solution, and the PH values of the solutions were adjusted to be 9, 10 and 11. The above mixed solution was transferred to a 100 mL autoclave, sealed, and placed in a constant temperature drying oven at 120 °C for 8 h. It was cooled to room temperature, washed several times with deionized water and absolute ethanol, and placed in a constant temperature drying oven at 60 °C to be dried. We named the samples synthesized under the above three different PH values ZnO-1, ZnO-2, and ZnO-3.

### 2.2. Characterization

X-ray powder diffraction (XRD) patterns were obtained from D8 Advance diffractometer with Cu K α radiation (λ = 0.15418 nm, Bruker, Karlsruhe, Germany). The Fourier transform-infrared (FT-IR) spectrum was investigated by a Bruker-V Vertex 70 (Bruker, Karlsruhe, Germany). A scanning electron micrograph (SEM) was obtained by a S-4800 scanning electron microscope (Hitachi, Tokyo, Japan). The elemental composition of the prepared samples was analyzed by X-ray photoelectron spectroscopy (XPS) on a Thermo ESCALAB 250Xi X-ray photoelectron spectrometer with monochromatic Al K Alpha radiation as an excitation source (Thermo Fisher Scientific, Waltham, Massachusetts, USA).

### 2.3. Preparation of ZnO Humidity Sensor

**Figure 1:**
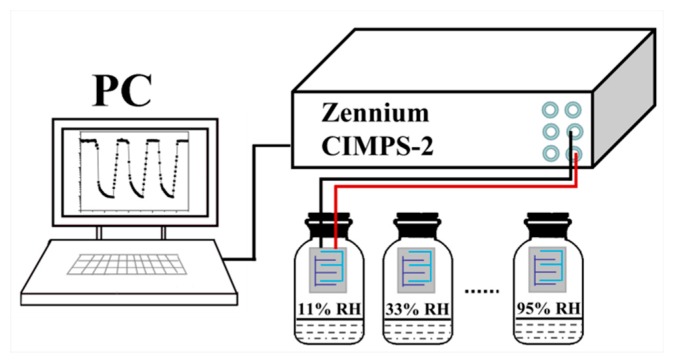
Schematic diagram of humidity experimental test device for ZnO humidity sensors.

The characteristic curve of humidity response was measured by a Zennium workstation (CIMPS-2, Zahner, Kronach, Germany). Different humidity conditions were established using several saturated solutions, including LiCl, MgCl_2_, Mg(NO_3_)_2_, NaCl, KCl, and KNO_3_, and the relative humidity (RH) in the container was 11%, 33%, 54%, 75%, 85%, or 95% for each of the above saturated solutions. During the test, ZnO powder and deionized water were mixed and sprayed on the Ag–Pd interdigital electrode, and the interdigital electrode was dried at 60 °C for 1 h. Then, the ZnO sensor was placed in an environment with each of the above RHs for measurement. The instrument voltage was set to AC 1 V during the test and the frequency was 40 Hz to 100 kHz. All performance tests were performed at room temperature (25 °C). Figure 1 shows a schematic illustration of the experimental setup.

## 3. Results and Discussion

**Figure 2:**
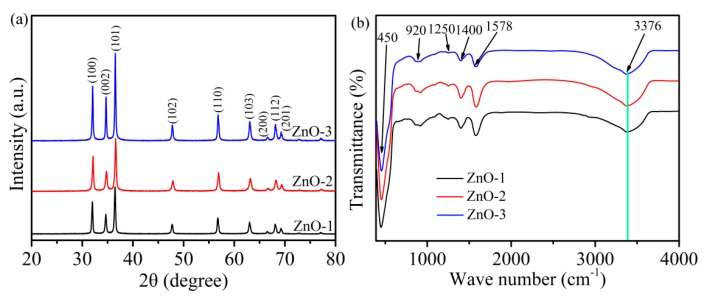
(**a**) X-ray powder diffraction (XRD) and (**b**) Fourier transform-infrared (FTIR) patterns of ZnO-1, ZnO-2, and ZnO-3.

The crystal structures and orientations of ZnO-1, ZnO-2, and ZnO-3 were investigated by XRD, as shown in Figure 2a. The diffraction peaks of all the samples were observed at 2θ of 31.96°, 34.58°, 36.43°, 47.67°, 56.78°, 62.98°, 66.51°, 68.01°, and 69.20°, and the corresponding lattice planes were (100), (002), (101), (102), (110), (103), (200), (112), (201), which is consistent with the typical hexagonal wurtzite structure of ZnO (JCPDS No. 36-1451) [25]. All the peaks were clear and sharp, indicating that the synthesized ZnO had good crystallinity. Compared with ZnO-1 and ZnO-2, the intensities of all the diffraction peaks of ZnO-3 were significantly enhanced, which means that ZnO-3 had the highest crystallinity and the largest grain size.

The effect of pH value of precursor on the functional groups contained in ZnO could be observed by FT-IR spectroscopy (Figure 2b). It can be seen that ZnO-1, ZnO-2, and ZnO-3 had similar FT-IR spectra. The peak which appeared at 450 cm^−1^ was attributed to the lattice metal oxygen cursor bond (Zn-O). The absorption bands at 920, 1250, 1400, and 1578 cm^−1^ were attributed to the symmetric and asymmetric tensile vibration of carboxyl group (COO^−^) of the acetate ion. The absorption peaks at about 3100–3600 cm^−1^ corresponded to the O-H tensile vibration of surface adsorption (–OH) [26]. It can be observed that the absorption peak of the O-H stretching vibration of ZnO slightly shifted toward a low wave number with the increase in the pH of the precursor, which was mainly due to the fact that a large amount of hydroxyl groups (–OH) are generated when the precursor solution of ZnO is alkaline. Moreover, hydroxyl group is a hydrophilic functional group which can adsorb a large amount of water molecules to enhance the adsorption process of the sensor.

**Figure 3:**
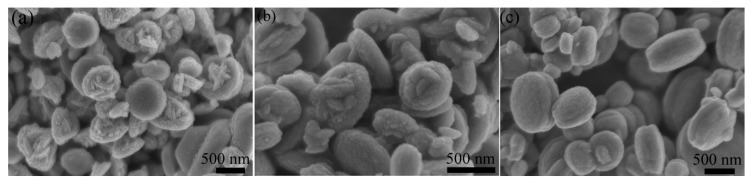
Scanning electron micrograph (SEM) images of (**a**) ZnO-1, (**b**) ZnO-2, and (**c**) ZnO-3.

In order to study the effect of pH on morphology of ZnO, the surface morphology of ZnO-1, ZnO-2, and ZnO-3 was characterized by SEM. All the samples showed a regular disc structure, as in Figure 3. In Figure 3a, ZnO-1 exhibited a regular disc structure with uniform distribution, most of which had a particle size about 500 nm. As shown in Figure 3b, ZnO-2 also exhibited a uniformly distributed disc structure with a particle size about 300 nm, which made the surface of the sample rough due to the corrosive action of the alkaline precursor solution. This structure had a larger specific surface area, which further strengthened the electrolytic conduction process, thereby increasing the response time of the sensor. As shown in Figure 3c, ZnO-3 showed a large particle size disc structure with a diameter about 1 μm. Compared with ZnO-1 and ZnO-2, the particle size of ZnO-3 is obviously increased, and the specific surface area was smaller, which seriously limits the application in the field of humidity sensors.

**Figure 4:**
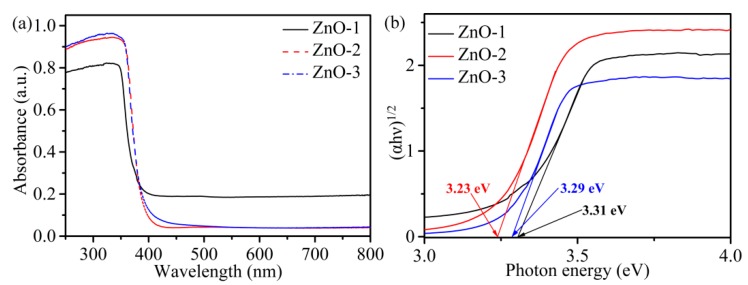
(**a**) UV-vis absorption spectrum and (**b**) band gap energy of ZnO-1, ZnO-2, and ZnO-3.

The optical properties of ZnO were studied by UV-visible diffuse reflectance spectroscopy, as shown in Figure 4. In Figure 4a, there is a significant absorption peak at nearly 340 nm for all samples, which corresponds to the band gap absorption of ZnO. Compared with ZnO-1, the absorption band edges of ZnO-2 and ZnO-3 were red-shifted, indicating that ZnO-2 and ZnO-3 had lower band gap energy [27]. The band gap energy of ZnO was calculated by the Kubelka–Munk function: (αhν)^2^ = A(hν-E_g_), where α, hν, E_g_, and A are the absorption coefficient, photon energy, band gap energy, and constant, respectively. It can be clearly seen in Figure 4b that the band gap energies of ZnO-1, ZnO-2, and ZnO-3 were 3.31, 3.23, and 3.29 eV, respectively. This indicates that the pH value of the solution affected the change in ZnO band gap energy. ZnO-2 had the lowest band gap energy, which indicates that it generated the minimum energy required for intrinsic excitation and it would be beneficial to improve the conductivity of ZnO-2 sensors.

**Figure 5:**
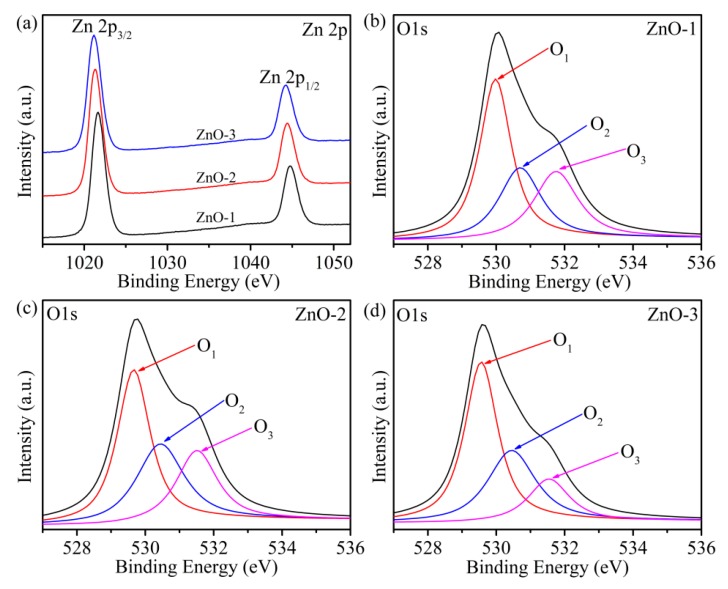
(**a**) X-ray photoelectron spectroscopy (XPS) spectra of Zn 2p of all samples; XPS spectra of O 1s of (**b**) ZnO-1, (**c**) ZnO-2, and (**d**) ZnO-3.

Figure 5 shows the chemical composition and valence state of the elements in ZnO-1, ZnO-2, and ZnO-3 characterized by XPS. The XPS spectrum of the prepared ZnO consisted of zinc (Zn 2p) and oxygen (O 1s). As can be seen from Figure 5a, all of the samples showed two peaks at binding energies of 1021.7 eV and 1044.8 eV, which correspond to Zn 2p_3/2_ and Zn 2p_1/2_, indicating that Zn exists in the Zn^+2^ state. Figure 5b–d shows the O 1s spectrum of ZnO-1, ZnO-2, and ZnO-3, in which three peak positions were fitted at binding energy 529.66, 530.44, and 531.51 eV, which correspond to lattice oxygen (O_1_), oxygen vacancies (O_2_), surface OH^−^ and the chemical adsorption of oxygen (C–O) (O_3_), respectively. The contents of O_2_ in ZnO-1, ZnO-2, and ZnO-3 were calculated to be 27.2%, 31.9%, and 32.6%, respectively, which indicates that the higher the PH value is, the easier the oxygen vacancies are formed on the ZnO surface. Rich surface oxygen vacancies can accelerate the decomposition of H_2_O molecules and provide more conductive ions (H_3_O^+^), which in turn improves the performance of ZnO humidity sensors.

**Figure 6:**
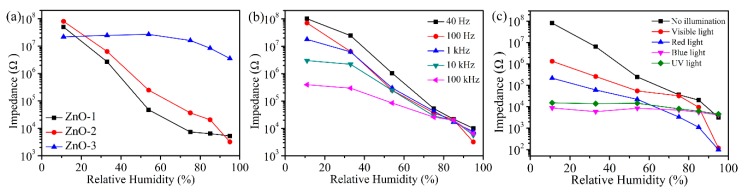
(**a**) Resistance response curve of ZnO humidity sensor in different humidity environments; (**b**) relationship between the resistance and RH of the ZnO-2 humidity sensor with an operating frequency of 40–100 kHz; (**c**) resistance relative humidity curve of ZnO-2 under different illuminations.

Figure S1 shows a current–voltage (I-V) curve of a ZnO-2 humidity sensor at different relative humidities. An increase in RH will increase the current of the ZnO-2 humidity sensor. When RH is 95%, the current of the ZnO-2 humidity sensor was increased by about 4 times at a given voltage. Based on the I-V curve of the ZnO-2 humidity sensor, the maximum output current point (at bias 1 V) was selected for subsequent tests. The changes in resistance of the ZnO humidity sensor in the range from 11% to 95% RH are shown in Figure 6a with a test frequency of 100 Hz. It can be seen that the ZnO-2 humidity sensor had a resistance change of more than four orders of magnitude and had a good linearity. The response of the humidity sensor is R = (R_11_ – R_95_)/R_95_ × 100%, where R_11_ is the resistance of 11% RH, and R_95_ is the resistance of 95% RH. From this, the response of ZnO-1, ZnO-2, and ZnO-3 humidity sensors could be calculated to be 954,696, 2,515,127 and 517%. The ZnO-2 sensor exhibited a high sensitivity mainly due to the presence of a large number of both hydroxyl groups and oxygen vacancies on its surface. The hydroxyl group is a hydrophilic functional group which makes ZnO-2 adsorb a large amount of water molecules and accelerates the adsorption speed of the sensor. In addition, a large number of oxygen vacancies can accelerate the dissociation process of water molecules, producing a large amount of conductive ions (H_3_O^+^). The combination of these two factors enhanced the performance of the ZnO-2 humidity sensor. The sensitivity of the ZnO-1 sensor was slightly lower than that of ZnO-2, mainly because the surface hydroxyl and oxygen vacancies were lower, which limited the adsorption and dissociation process. The ZnO-3 sensor had no response over the entire humidity range, mainly because the limited specific surface area of ZnO-3 limited electrolytic conduction. Moreover, the oxygen vacancies on the surface of ZnO-3 may shift to the inside when pH = 11, thus reducing the dissociation ability to H_2_O.

To determine the best test frequency for ZnO-2 humidity sensor, we measured the resistance of the sensor at 40, 100, 1000, 10,000, and 100,000 Hz with different RH, as in Figure 6b. It can be seen that the linearity of the curve was poor at 40 Hz, and the sensitivity of the sensor was lower at 1000, 10,000, and 100,000, which is due to the fact that H_2_O is not easily polarized at high frequencies. Compared to the other samples, the ZnO-2 humidity sensor exhibited both good linearity and high sensitivity at 100 Hz, so the best test frequency for the ZnO-2 sensor was 100 Hz and all subsequent performance tests were performed at 100 Hz.

In order to investigate the effect of illumination on the ZnO-2 humidity sensor, the changes in resistance under white light, red light, blue light, and UV light (395 nm) were tested. It can be observed that ZnO-2 did not have any change in resistance over the entire RH range under high energy (UV and blue light) illumination, which is due to ZnO-2 acquiring high energy, and photoelectrons play a major role in conducting electricity. Although the ZnO-2 humidity sensor can change by four orders of magnitude over the entire RH range under white and red light illumination, the linearity is poor. Therefore, ZnO-2 humidity sensor achieves both the highest sensitivity and the best linearity when there is no above illuminations, which expands the working range and reduces the production cost of the sensor.

**Figure 7:**
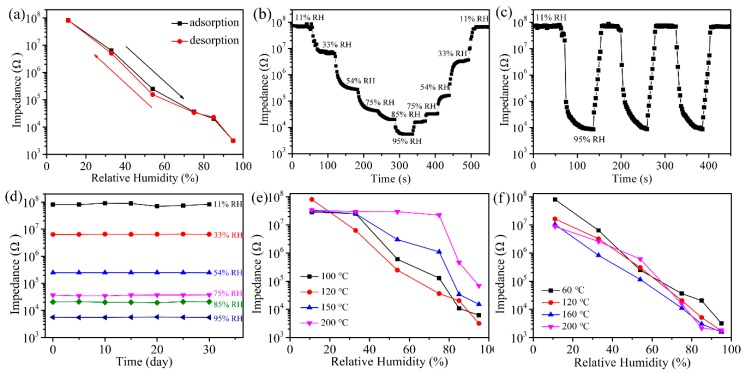
(**a**) Hysteresis characteristics, (**b**) response to different relative humidity levels, (**c**) response and recovery performance, and (**d**) long-term stability based on the ZnO-2 humidity sensor with RH from 11% to 95% at 100 Hz; (**e**) resistance response curves of ZnO-2 sensors with synthesis temperatures of 100 °C, 120 °C, 150 °C, and 200 °C; (**f**) resistance response curves of ZnO-2 sensors with drying temperatures of 60 °C, 120 °C, 160 °C, and 200 °C.

Humidity hysteresis is the biggest difference between the adsorption and desorption process of humidity sensors, and it is used to detect the reliability of humidity sensors. The hysteresis of the ZnO-2 humidity sensor is shown in Figure 7a. RH takes values from 11% to 95% in the adsorption process and vice versa in the desorption process, the resistance is always bigger in the whole adsorption process than that in the desorption process. The calculated maximum hysteresis error (γH) of the ZnO-2 sensor in the range of 11–95% RH was 0.9%, indicating that ZnO-2 humidity sensor has good reliability (γH = ± ΔH_max_/2F_FS_, where ΔH_max_ is the maximum hysteresis value and F_FS_ is the full-scale output) [28].

The response to change in ambient humidity is an important manifestation of the performance of a humidity sensor. Figure 7b shows the typical time dependence of the ZnO-2 humidity sensor from low–high–low. It can be seen that the difference between the rising and the falling curves was very small, indicating that ZnO-2 humidity sensor has good reproducibility. Response/recovery time and repeatability are also important parameters for evaluating the performance of a humidity sensor. Figure 7c shows the repeatability and response/recovery time of the sensor over three adsorption and desorption cycles from 11% to 95% RH. It can be observed that the response of the ZnO-2 sensor was consistent in three adsorption and desorption cycles, indicating that the ZnO-2 humidity sensor had good repeatability. It is well known that the time taken for a sensor to reach 90% of the total impedance change is defined as the response or recovery time [29]. The response/recovery time of the ZnO-2 humidity sensor was 31/14 s. The ZnO-2 sensor had small hysteresis and a fast response/recovery time, mainly because of the large amount of hydrophilic functional groups (OH^−^), large specific surface area, and rich oxygen vacancies on the surface of it, which enhance the electrolysis and conduction processes. Compared to the humidity sensor performance reported in previous work (Table 1), the ZnO-2 sensor had a high response, fast response/recovery speed, and small hysteresis.

Figure 7d shows the stability of the ZnO-2 sensor from 11% to 95% RH, measured every 5 days for a total of 30 days. It can be found that the sensor had slight fluctuations in resistance only at 11% RH and it was stable from 33% to 95% RH. So, the ZnO-2 sensor has good long-term stability. All the above results show that ZnO-2 is a high-performance practical humidity sensor. In order to investigate the effect of ZnO-2 synthesis temperature and drying temperature on sensor performance, we measured the resistance response curves of ZnO-2 humidity sensors at different temperatures. In Figure 7e,f, it can be observed that the synthesis temperature and the drying temperature had a large influence on the response of the ZnO-2 humidity sensor. It is apparent that when the synthesis temperature was 120 °C and the drying temperature was 60 °C, the sensitivity and linearity of the sensor were superior to other conditions.

**Figure 8:**
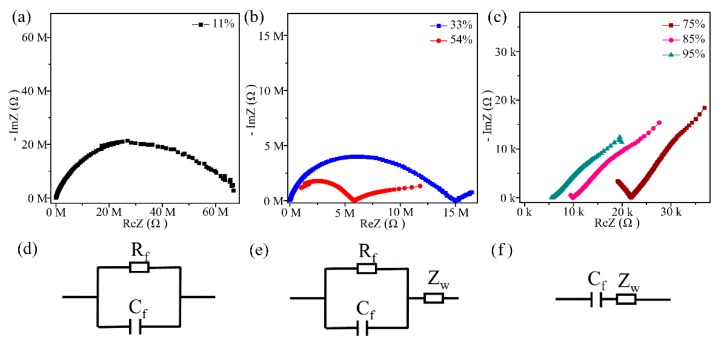
(**a**–**c**) ZnO humidity sensor has a complex impedance spectroscopy of 11% to 95% RH and (**d**–**f**) corresponding equivalent circuit (R_f_: film resistance; C_f_: film impedance; Z_w_: Warburg impedance).

In order to study the mechanism of humidity sensitivity, complex impedance spectroscopy (CIS) and equivalent circuit (EC) were used to explain the conduction process of ZnO sensor. The test frequency was from 40 Hz to 100 kHz and the test voltage was AC 1V. At low humidity (11% RH), the CIS curve showed a semicircle (Figure 8a), and the EC can be described by a parallel connection of a resistor (R_f_) and a capacitor (C_f_) (Figure 8d) [36,37]. Since a small amount of water molecules are adsorbed on the surface of ZnO by chemisorption, the coverage of water on surface of ZnO is not continuous, and the conductive process may be conductive by the OH^−^ promoting hopping of a small amount of protons on the surface of ZnO, resulting in high impedance. With a further increase in RH (33%, 54% RH), the CIS curves showed that the semicircle gradually became smaller, and a short line appeared in the semicircular tail in the low frequency range (Figure 8b). The EC can be assembled as the parallel of the capacitor and the resistor to be in series with the Warburg impedance (Z_w_) (Figure 8e). Z_w_ represents the short-line portion of the CIS, and Z_w_ plays a major role in the impedance response of ZnO [38]. At this stage, mainly due to the action of Z_w_, H^+^ ions and hydronium ions (H_3_O^+^) diffuse in ZnO to conduct electricity [39]. At high humidity (75%, 85%, 95% RH), the CIS curve gradually became a straight line in the low frequency region (Figure 8c), and the EC had C_f_ and Z_w_ connected in series (Figure 8f). At this time, with the increase of adsorbed water molecules, the ion transport mechanism was formed (Grothuss). H_3_O^+^ ions play a major role in the entire conduction process [3,40].

**Figure 9:**
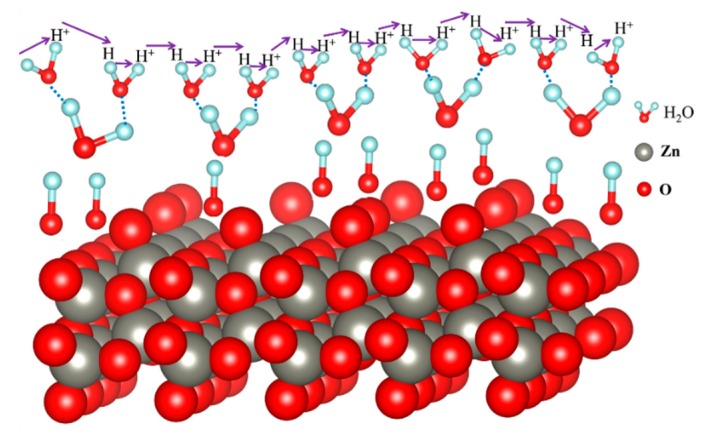
Theoretical model for adsorbing water on the surface of the ZnO-2 humidity sensor.

The humidity sensing characteristics of the ZnO-2 humidity sensor depend on the adsorption and the desorption capabilities of H_2_O molecule. Figure 9 shows the sensible humidity mechanism of the ZnO-2 humidity sensor, and it exhibited excellent performance mainly due to rich surface oxygen vacancies and hydroxyl groups. The presence of surface oxygen vacancies and hydroxyl groups on the surface of ZnO-2 was also verified from the analysis of FT-IR and XPS. Among them, the hydroxyl group belongs to the relative functional group, which can adsorb a large amount of H_2_O molecules. The oxygen vacancies can accelerate the decomposition of H_2_O molecules, thereby improving the performance of the sensor. At low humidity (11% RH), a small number of H_2_O molecules are adsorbed on the hydroxyl groups by hydrogen bonding to form a single water molecule layer or a few water molecule layers. At this time, a small amount of H_2_O is decomposed into H^+^ and OH^−^ by oxygen vacancies on the surface of ZnO-2, where conduction mainly depends on H^+^ and proton conduction is discontinuous. With the increase in water molecules (33%, 54% RH), more water molecules are adsorbed on the surface of ZnO-2 to make proton conduction easier, and H^+^ and H_2_O combine to form H_3_O^+^. At this stage, H^+^ and H_3_O^+^ participate in conduction. At high humidity (75%, 85%, 95% RH), a large number of H_2_O molecules form a continuous water film on the surface of ZnO-2, and H_3_O^+^ ions play a leading role in the whole process. Moreover, the large specific surface area of ZnO-2 can adsorb more water molecules to accelerate the ion conduction process, which is also an important factor to enhance the performance of the sensor. The desorption process of the ZnO-2 humidity sensor is relatively simple. When ZnO-2 is transferred from a high humidity to low humidity environment, the adsorption of water molecules on the surface of ZnO-2 will be released immediately. Since the ZnO-2 is uniformly distributed and has a large specific surface area, the desorption process of water molecules will not be delayed. Therefore, the sensor shows a fast recovery speed and low hysteresis.

## 4. Conclusions

In summary, a low-cost and high-performance ZnO resistive humidity sensor was prepared using a simple and efficient one-step synthesis method. The experimental results showed that the PH value of precursor solution affects the performance of ZnO humidity sensors. A large number of hydroxyl groups (OH^−^) and rich oxygen vacancy defects are generated on the surface of ZnO when the PH value of the precursor solution is 10. Rich oxygen vacancies on the surface of ZnO can decompose more water molecules to produce a large number of conductive ions (H_3_O^+^), which enhances the conductivity of the sensor. Furthermore, the hydrophilic functional groups (OH^−^) on ZnO can adsorb a large number of water molecules, and the large specific surface area of ZnO can accelerate the ion (H_3_O^+^) conduction process, which makes the ZnO sensor exhibit an excellent humidity performance. Such a ZnO humidity sensor exhibits high sensitivity (14,415), good linearity, small hysteresis (0.9%), fast response/recovery time (31/15 s), and stability in the range from 11% to 95% RH. 

## Figures and Tables

**Table 1:** The performance of the ZnO-2 humidity sensor compared to previous published work.

Material	Order of Impedance Change	Response Time (s)	Recovery Time (s)	Hysteresis (%)	Ref.
Er-ZnO	3	32.3	39.6	—	[30]
ZnO-SiO_2_	4	50	100	2	[31]
Sulfonated Poly	4	100	105	3	[32]
CdS/ZnO	—	110	32	5.4	[33]
TiO_2_ nanotube	2	100	190	—	[34]
ZnO nanostructure	—	90	120	—	[35]
ZnO	4	31	15	0.9	This work

## Supplementary Materials

The following are available online at https://www.mdpi.com/1424-8220/19/23/5267/s1, Figure S1: I-V curve of ZnO-2 humidity sensor in 11, 75, 95% RH environment.
