# Supplementary material for: Effects of pH on High-Performance ZnO Resistive Humidity Sensors Using One-Step Synthesis
Source: Sensors (Basel). 2019 Nov 29;19(23):5267. doi: 10.3390/s19235267 (PMC6929030; doi:10.3390/s19235267)
Supplement: Supplementary file 1 [file sensors-19-05267-s001.pdf]

Supplementary Materials

Effects of pH on high performance ZnO resistive humidity sensors using one-step synthesis

Shuguo Yu, Hongyan Zhang*, Jun Zhang* and Zhijun Li

School of Physical Science and Technology, Xinjiang University, Urumqi 830046, China;
yushuguo0818@163.com (S.Y.); lizhjun@xju.edu.cn (Z.L.)
*Correspondence: zhy@xju.edu.cn (H.Z.); zhj@xju.edu.cn (J.Z.)

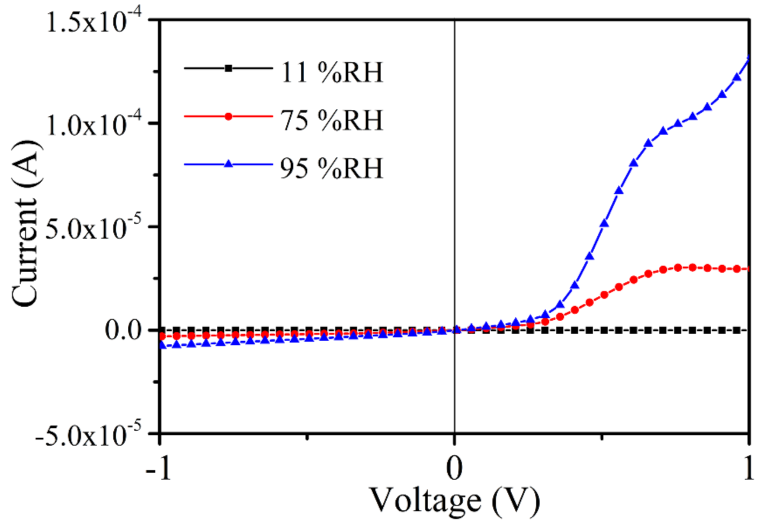

Figure S1. I-V curve of ZnO-2 humidity sensor in 11, 75, 95 %RH environment.
